# Optical Genome Mapping Enhances Structural Variant Detection and Refines Risk Stratification in Chronic Lymphocytic Leukemia

**DOI:** 10.3390/genes17010106

**Published:** 2026-01-19

**Authors:** Soma Roy Chakraborty, Michelle A. Bickford, Narcisa A. Smuliac, Kyle A. Tonseth, Jing Bao, Farzana Murad, Irma G. Domínguez Vigil, Heather B. Steinmetz, Lauren M. Wainman, Parth Shah, Elizabeth M. Bengtson, Swaroopa PonnamReddy, Gabriella A. Harmon, Liam L. Donnelly, Laura J. Tafe, Jeremiah X. Karrs, Prabhjot Kaur, Wahab A. Khan

**Affiliations:** 1 Division of Hematopathology, Department of Pathology and Laboratory Medicine, Dartmouth Hitchcock Medical Center, Lebanon, NH 03756, USA; 2 Geisel School of Medicine, Dartmouth College, Hanover, NH 03755, USA; 3 Laboratory for Clinical Genomics and Advanced Technology (CGAT), Department of Pathology and Laboratory Medicine, Dartmouth Hitchcock Medical Center, Lebanon, NH 03756, USA; 4 Department of Hematology/Oncology, Dartmouth Hitchcock Medical Center, Lebanon, NH 03756, USA

**Keywords:** optical genome mapping, chronic lymphocytic leukemia, time-to-first treatment, structural variants, next-generation sequencing, FISH, IGHV mutation status

## Abstract

**Background:** Optical genome mapping (OGM) detects genome-wide structural variants (SVs), including balanced rearrangements and complex copy-number alterations beyond standard-of-care cytogenomic assays. In chronic lymphocytic leukemia (CLL), cytogenetic and genomic risk stratification is traditionally based on fluorescence *in situ* hybridization (FISH), karyotyping, targeted next-generation sequencing (NGS), and immunogenetic assessment of immunoglobulin heavy chain variable region (IGHV) somatic hypermutation status, each of which interrogates only a limited aspect of disease biology. **Methods:** We retrospectively evaluated fifty patients with CLL using OGM and integrated these findings with cytogenomics, targeted NGS, IGHV mutational status, and clinical time-to-first-treatment (TTFT) data. Structural variants were detected using OGM and pathogenic NGS variants were derived from a clinical heme malignancy panel. Clinical outcomes were extracted from the electronic medical record. **Results:** OGM identified reportable structural variants in 82% (41/50) of cases. The most frequent abnormality was del(13q), observed in 29/50 (58%) and comprising 73% (29/40) of all OGM-detected deletions with pathologic significance. Among these, 12/29 (42%) represented large *RB1*-spanning deletions, while 17/29 (58%) were focal deletions restricted to the miR15a/miR16-1 minimal region, mapping to the non-coding host gene *DLEU2*. Co-occurrence of adverse lesions, including deletion 11q/*ATM*, *BIRC3* loss, trisomy 12, and deletion 17p/*TP53*, were recurrent and strongly associated with shorter TTFT. OGM also uncovered multiple cryptic rearrangements involving chromosomal loci that are not represented in the canonical CLL FISH probe panel, including *IGL::CCND1*, *IGH*::*BCL2*, *IGH*::*BCL11A*, *IGH*::*BCL3*, and multi-chromosomal copy-number complexity. IGHV data were available in 37/50 (74%) of patients; IGHV-unmutated status frequently co-segregated with OGM-defined high-risk profiles (del(11q), del(17p), trisomy 12 with secondary hits, and complex genomes whereas mutated IGHV predominated in OGM-negative or structurally simple del(13q) cases and aligned with indolent TTFT. Integration of OGM with NGS further improved genomic risk classification, particularly in cases with discordant or inconclusive routine testing. **Conclusions:** OGM provides a comprehensive, genome-wide view of structural variation in CLL, resolving deletion architecture, identifying cryptic translocations, and defining complex multi-hit genomic profiles that tracked closely with clinical behavior. Combining OGM and NGS analysis refined risk stratification beyond standard FISH panels and supports more precise, individualized management strategies in CLL. Prospective studies are warranted to evaluate the clinical utility of OGM-guided genomic profiling in contemporary treatment paradigms.

## 1. Introduction

Chronic lymphocytic leukemia (CLL) is a genomically heterogeneous lymphoid malignancy characterized by recurrent structural variants (SVs), copy-number alterations, and gene-sequence changes that influence clinical behavior, prognostics, and therapeutic response. CLL is managed in an era where genomic information directly informs when to treat, how intensively to treat, and how closely to monitor patients, rather than serving solely as a diagnostic label. Recurrent cytogenetic abnormalities such as 13q14 locus deletion, trisomy 12, 11q/*ATM* locus deletion, and 17p/*TP53* locus deletion, together with immunogenetic markers like IGHV, are embedded in contemporary prognostic schemes and NCCN-endorsed risk-adapted algorithms because they track with time-to-first-treatment, response to chemoimmunotherapy, and long-term survival [1]. For example, isolated focal deletion 13q is typically associated with indolent disease and pro-longed observation, whereas deletion 11q and deletion 17p often associate with earlier treatment, treatment resistance, and inferior outcomes. Cytogenomic abnormalities, including del(13q14), trisomy 12, del(11q/*ATM*), and del(17p/*TP53*), are a key component of current prognostic models and risk-adapted treatment algorithms [1,2,3]. Among these, del(13q) is the most frequent aberration and is typically associated with favorable outcomes when present as an isolated lesion [4,5]. However, growing evidence indicates that the size and gene content of del(13q), particularly involvement of RB1, and co-occurring genomic complexity can modify its clinical implications [4,5,6].

In parallel, IGHV somatic hypermutation status has emerged as a powerful independent predictor of outcomes in CLL. Patients with mutated IGHV generally experience indolent disease with prolonged survival, whereas unmutated IGHV is associated with aggressive clinical course, early treatment requirement, and inferior response to conventional chemoimmunotherapy [7,8]. Contemporary risk models therefore incorporate both IGHV status and genetic features, reflecting the interplay between cell-of-origin biology and acquired genomic damage.

Parallel advances in sequencing have underscored that gene-level mutations and larger structural or cytogenetic events represent complementary layers of risk rather than interchangeable findings. A useful cross-disease illustration has come from acute myeloid leukemia, where distinct classes of *IDH1* and *IDH2* mutations with similar molecular targets have been shown to confer markedly different patterns of response and survival, underscoring that not all mutations within a given gene family are prognostically equivalent [9]. Analogously, in CLL, seemingly similar categories of genetic alterations, such as 13q deletions or TP53 pathway disruption, may have very different clinical implications depending on their structural context and co-occurring genomic lesions; motivating a more granular approach to risk stratification. Sequence variants in genes such as *TP53*, *NOTCH1*, and *BIRC3* can modify prognosis even within the same cytogenetic category, while genomic complexity and multi-hit chromosomal structural events add an additional dimension that is not captured by isolated lesions alone. Outcome-focused leukemia studies have shown that even closely related molecular alterations can drive divergent clinical trajectories when considered in their broader genomic context, highlighting the need to move beyond binary “mutation present/absent” frameworks toward integrated models that incorporate both sequence and genomic structural information. In this study, we use OGM to digitally resolve the structural variant landscape in CLL and integrate these findings with NGS and IGHV status to examine how different patterns of genomic disruption relate to time-to-first-treatment (TTFT) and clinically relevant risk stratification.

To elaborate further on legacy assays, FISH panels detect canonical CLL lesions but interrogate only a limited set of loci. Karyotyping can have limited sensitivity in CLL due to mitotic inactivity and low resolution, and exome-based NGS panels can detect sequence-level variants but provide little information about chromosomal complexity. Consequently, clinically relevant abnormalities, including cryptic translocations, balanced rearrangements, complex chromosomal patterns, and multi-locus deletions, may remain undetected using current routine methods [10].

We utilized OGM, a genomic tool capable of detecting genome-wide SVs with high resolution, to CLL biology. Its performance has been validated in multiple hematologic malignancies, revealing abnormalities that frequently escape detection by FISH or karyotype [11,12].

Specifically, we apply OGM to a fifty-patient CLL clinical cohort to assess its clinical impact by defining the structural variant landscape and catalog high vs. low impact chromosomal lesions in CLL biology. We further integrate OGM results with IGHV mutation, FISH, and NGS status and correlate these composite genomic features with TTFT.

## 2. Materials and Methods

### 2.1. Patient Cohort and Study Design

This retrospective cohort included 50 patients with confirmed CLL who underwent clinical genomic testing at our institution; including 2 patients with high-count monoclonal B-cell lymphocytosis (MBL) and one case later reclassified as mantle cell lymphoma (MCL). Patients were identified through electronic medical record review, with cytogenetic, NGS, OGM, and IGHV data collated alongside clinical variables (age at diagnosis, sex, diagnosis date, treatment history, and TTFT); structural and sequence variants were classified using AMP/ClinGen/CGC somatic criteria (tier 1—pathogenic, tier 2—likely pathogenic), excluding tier 3/4 variants. The study was conducted under institutional IRB approval (STUDY02002136), and all testing was performed in CLIA-certified laboratories. These tiering thresholds were chosen to focus on alterations with the strongest evidence for clinical relevance in OGM analysis workup; however, this approach necessarily underrepresents potentially informative Tier 3 SV/copy number (CN) events of uncertain significance and may underestimate the contribution of lower-evidence variants to genomic complexity. As such, the structural and mutational profiles described here should be interpreted as conservative estimates of clinically validated risk rather than an exhaustive catalog of all detectable genomic changes. 

Although the cohort was anchored in clinically diagnosed CLL, we retained two MBL cases and one case later reclassified as MCL in the genomic analyses because they underwent the identical cytogenomic workflow. This choice slightly broadens the biological spectrum represented and may introduce limited heterogeneity at the disease-boundary, but it allows a more complete appraisal of how OGM performs in a real-world diagnostic setting where such borderline and reclassified cases are encountered.

### 2.2. Sample Types and OGM Workflow

Peripheral blood (PB) and bone marrow (BM) samples collected in EDTA or sodium heparin were used for OGM procurement. Ultra-high molecular weight DNA was isolated with Bionano Prep SP procurement kits, quantified by Qubit dsDNA assays, and processed on the Bionano Saphyr system, where labeled long DNA molecules were linearized in nanochannels, imaged to generate single-molecule genome maps, and analyzed with Bionano Solve using the rare variant and guided analysis pipelines optimized for somatic SV detection. Structural variants were tiered by pipeline output. Tier 1 (pathogenic with strong support) and tier 2 (likely pathogenic with moderate support) calls were included in downstream analyses.

### 2.3. Additional Cytogenomics Work-Up

Clinical interphase FISH was performed on peripheral blood or bone marrow using a laboratory-validated CLL panel targeting 13q14 (D13S319), 11q22.3 (*ATM*), 17p13.1 (*TP53*), *IGH*::*CCND1* loci, and trisomy 12 (Abbott Molecular). A total of 200 interphase nuclei were analyzed per case per probe on the panel by two microscopists. Cases without available FISH results were designated as not available (N/A) for concordance analyses.

### 2.4. Next-Generation Sequencing and IGHV Somatic Hypermutation Analysis

Genomic DNA was extracted using automated EZ1-based workflows (Qiagen, Hilden, Germany) as part of routine clinical laboratory processing. NGS was performed using a clinical hematologic malignancy panel virtually sliced from an Agilent Exome v8 backbone and sequencing data processed using an in-house bioinformatics suite (AUGMET), as previously validated for routine clinical application [13]. IGHV mutational status was detected by NGS-based sequencing and alignment to germline IGHV reference sequences. It was noted as mutated, unmutated, or not available for correlation with OGM-defined structural variant categories and TTFT.

### 2.5. Integrative Genomic Analyses Approach

To assess integrative genomic patterns, OGM findings were compared with canonical CLL abnormalities detected by clinical FISH, single-nucleotide, and indel variants from NGS, IGHV mutation status, established cytogenetic risk profiles, and the published CLL genomic literature. FISH concordance for loci covered by the CLL panel was categorized as “full” (all FISH-detected lesions identified by OGM) or “additional” (all FISH-detected lesions identified by OGM with additional events detected by OGM). IGHV status and NGS variants were mapped onto OGM-defined structural rearrangements, including isolated/focal del(13q), *RB1*-spanning del(13q), del(11q), del(17p), trisomy 12, complex genomes, and OGM-negative genomes. Given the limited cohort size and partial availability of FISH, NGS, and IGHV data, these integrative categories are intended as preliminary frameworks to visualize genomic patterns rather than as formal, independently validated risk groups. The associations between specific chromosomal structural constellations and clinical behavior should therefore be viewed as hypothesis-generating and will require confirmation in larger, prospectively assembled cohorts.

### 2.6. Time-to-First-Treatment (TTFT) Assessment & Statistical Analysis

In this study, time-to-first treatment (TTFT) was defined as the interval from the date of CLL diagnosis to initiation of first-line therapy, with untreated patients censored at the date of last documented follow-up. Terms describing TTFT (e.g., shorter or longer) are used descriptively to reflect earlier versus delayed treatment initiation relative to the cohort distribution, rather than predefined categorical thresholds. TTFT distributions were estimated using Kaplan–Meier methods. Comparisons between OGM-defined genomic risk categories (e.g., moderate to low risk-focal del13q/OGM-negative vs. higher risk del11q, del17p/complex genomes) were performed using two-sided log-rank tests, with *p* values interpreted descriptively. Because TTFT data were available for only a subset of patients and event counts were modest, the study was not powered for definitive prognostic modeling or multivariable adjustment; statistical comparisons are presented to illustrate potential directional trends rather than to establish new clinical cutoffs. For the purposes of this analysis, complex genomes in CLL are defined *a priori* as those harboring multichromosomal distinct copy-number and/or structural/numerical abnormalities, consistent with current concepts of cytogenetic/genomic complexity in CLL. The ‘high-risk’ OGM group at a minimum comprised of cases with del(17p)/*TP53* loss; all other profiles, including isolated or focal del(13q), without extensive additional changes, and OGM-negative genomes, were classified as moderate/low-risk We additionally applied a nonparametric Mann–Whitney test to compare TTFT distributions. No formal adjustment for multiple comparisons was performed, as the TTFT analyses were restricted to a small number of pre-specified OGM-defined groupings. OGM-derived genomic groups were then examined for differences in TTFT in a descriptive fashion to explore associations between risk categories and early treatment requirements.

## 3. Results

### 3.1. Patient Demographics and Baseline Characteristics

The study cohort consisted of fifty patients who underwent cytogenetic and genomic testing. Baseline demographic and diagnostic characteristics are summarized in Table 1. The median age at diagnosis was 63 years for males and 65 years for females, with an equal sex distribution (25 males and 25 females). The majority of cases were diagnosed as CLL (47/50, 94%). Two patients met criteria for MBL, and one case was subsequently reclassified as MCL after OGM work-up. This case was previously reported following the identification of a balanced *IGL*::*CCND1* translocation by OGM [14].

### 3.2. Global Structural Variant Landscape Identified by OGM

**Figure 1:**
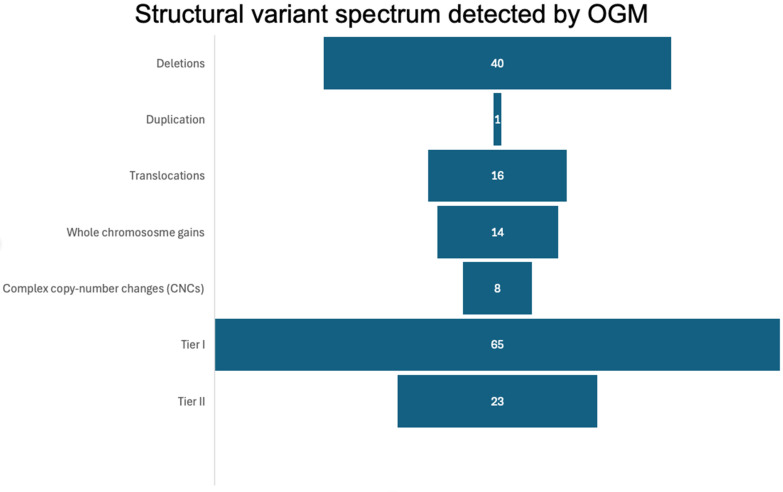
Structural variant spectrum detected by OGM in the CLL cohort. This horizontal bar graph displays the distribution of structural variant (SV) events detected by OGM, categorized by SV class. Deletions were the most frequent event type (*n* = 40), followed by translocations (*n* = 16), whole chromosome gains (*n* = 14), and complex copy-number changes (CNCs; *n* = 8). A single duplication event was identified. The total number of tier 1 (*n* = 65) and tier 2 (*n* = 23) events is shown to illustrate the overall burden of clinically relevant structural variants across the cohort. Counts reflect the number of events rather than the number of patients.

OGM identified reportable structural variants in 41 of 50 cases (82%), encompassing deletions, duplications, balanced and unbalanced translocations, and multi-chromosomal copy-number complexity, whereas 9 cases (18%) were OGM-negative. The distribution of tier 1/2 SV classes is summarized in Figure 1, with deletions representing the most frequent event type (40 events), followed by translocations (16 events), whole-chromosome gains (14 events), complex copy-number changes (8 events), and a duplication event. In aggregate, OGM detected 65 oncogenic (tier 1) and 23 likely oncogenic (tier 2) SVs, and many patients contributed multiple events, underscoring the high prevalence of multi-hit genomic architecture in this cohort. Compared with standard FISH, OGM showed high concordance (Table 2) for canonical CLL lesions (del(13q), del(11q), del(17p), and trisomy 12) while also revealing additional cryptic rearrangements and co-occurring abnormalities.

### 3.3. Deletion of 13q14 Presented as the Dominant Structural Variant with Heterogeneous Deletion Sizes

**Figure 2:**
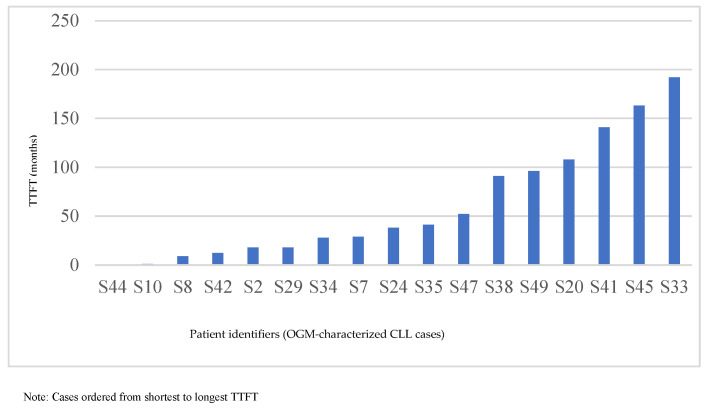
Bar plot of time-to-first treatment (TTFT, months) for CLL patients with available treatment initiation data. The X-axis shows individual patient identifiers, each representing a unique case with corresponding OGM-defined structural variants. The Y-axis depicts TTFT in months, with cases ordered from shortest to longest TTFT, illustrating a gradient from high-risk structural events requiring early therapy (e.g., S44, S10, and S8; see also Table 3 for details) to more indolent genomic profiles with prolonged observation (e.g., S45, S33). Table 3 provides detailed OGM findings for each patient and highlights structural variants that may contribute to these clinical differences.

OGM detected deletion of 13q as the most common abnormality in 29 of 50 patients (58%), accounting for 29 of 40 (73%) OGM-identified tier 1/2 deletions. Among these, 12 cases (42%) showed large deletions extending into *RB1* (S8, S13, S14, S15, S20, S23, S32, S35, S36, S39, S48, and S49—Table 2), whereas 17 (58%) had focal deletions restricted to the miR15a/miR16-1 (*DLEU2*) minimal deleted region; most were monoallelic, with one clearly biallelic case (S17) and one multi-locus event (S15), implicating 13q14 cytoband hotspot in translocation. Focal del(13q) was associated with longer TTFT and more indolent disease, whereas *RB1*-involving deletions often co-occurred with del(17p), del(11q), trisomy 12, or complex CNCs and were enriched in patients with shorter TTFT (e.g., S8, S14, S15, S20, and S35—Table 3, Figure 2). These data reinforce the prognostic relevance of del(13q) size and gene content.

### 3.4. Co-Occurring High-Risk Structural Abnormalities

High-risk structural lesions frequently co-occurred within individual patients, including del(11q) involving *ATM* and/or *BIRC3*, del(17p)/TP53 loss (S26, S34, S35, and S44) with *TP53* point mutations on the opposite allele. These events were also found in S5, S21—Table 2, further compounding risk. The deletion of 11q often appeared alongside del(13q) (S2, S38, and S46) and was associated with shorter TTFT (S2, S38). Deletion of 17p commonly coincided with multichromosomal CNCs and *TP53* sequence variants (S5, S21, S34, S35, and S44), reflecting marked genomic instability. Trisomy 12 co-occurred with *NOTCH1* variants (S12, S48, and S50) in our cohort, aligning with intermediate-to-adverse biology [15]. These findings illustrate the frequent convergence of multiple high-risk lesions within individual patients.

### 3.5. Rare CLL Rearrangements by OGM

**Figure 3:**
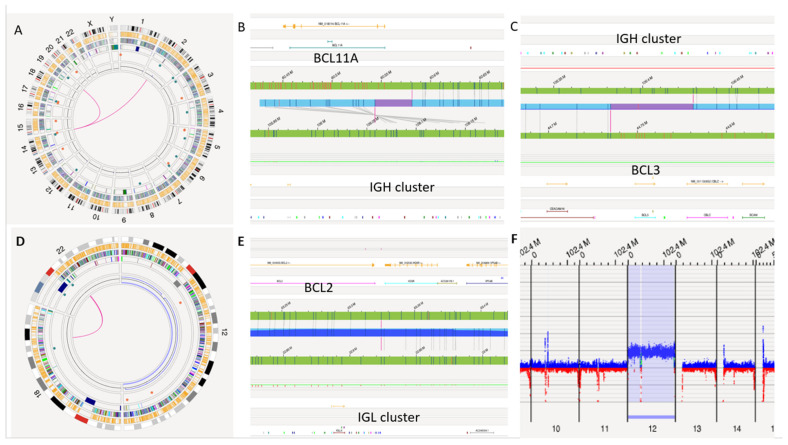
Chromosomal translocations involving the immunoglobulin locus. (**A**–**C**) Double chromosomal translocation at the *IGH* locus/14q32 involved chromosomes 2p16.1/*BCL11A* and 19q13.32/*BCL3* is shown in the circos plot (3A). The molecule map of each translocation is depicted in 3B and 3C. (**D**–**F**) *IGL*::*BCL2* translocation co-occurring with trisomy 12. Magenta lines depicting the translocation is shown in the circos plot (3D) and the OGM molecule map (3E). The whole genome view (3F) is shown next to these images.

**Figure 4:**
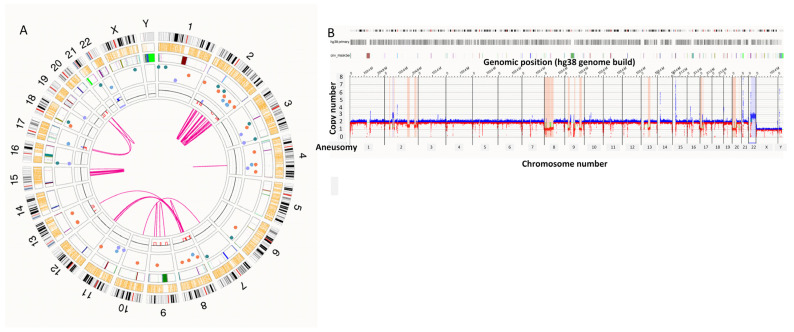
(**A**) CLL genomic complexity. Multiple chromosomal aberrancies comprising of translocations and intrachromosomal rearrangements detected by OGM across chromosomes 2, 8, 9, 13, 17, 20, and 22. (**B**) Whole genome view showing partial and full chromosome aneusomies (blue upwards shift is gain and red downward shift is loss).

OGM uncovered prognostically important chromosomal translocations involving the immunoglobulin locus. These events included a rare double-*IGH* rearrangement involving *IGH*::*BCL11A* and *IGH*::*BCL3* (S10; Figure 3A–C), a rare *IGL*::*BCL2* t(18;22) with gain of chromosome 12 (S37; Figure 3D–F), and an *IGH*::*BCL2* t(14;18) rearrangement (S45), which has been reported in approximately 1–2% of CLL cases overall [16]. Collectively, this demonstrated genome mapping’s ability to reveal rare immunoglobulin family of gene-related lesions with direct prognostic genome-wide impact. Further complex CNCs patterns were present in 8 of 41 (20%) patients. Prototypic examples included multi-hit 13q/21q/5q changes, broad copy number alterations affecting chromosomes 2, 8, and 9, and multichromosomal gains and losses with concurrent del(17p). These complex SV profiles, particularly in S34, S35 (Figure 4), and S44, were uniformly associated with adverse TTFT.

Although numerically rare, these structurally complex or atypical genomic profiles clustered among the patients with the shortest TTFT and most aggressive clinical courses, suggesting that they may represent a distinct subset rather than random outliers. Similar patterns have been described in other leukemias, where uncommon combinations of structural lesions, for example, t(6;9)(*DEK*::*NUP214*) concurrent with inv(16)(*CBFB*::*MYH11*) in chronic myeloid leukemia, have been linked to particularly unfavorable disease progression [17]. This reinforces the concept that specific architectural configurations can identify high-risk biology even when encountered infrequently.

### 3.6. Concordance Studies

FISH results were available for 49 of 50 patients (98%), with OGM yielding concordant findings for canonical CLL lesions in 45 of 49 (91.8%), while also detecting additional cryptic translocations, focal deletions, and complex CNCs in selected cases (e.g., S10, S33, S38, S44, and S48). NGS data were available for 29 of 50 patients (58%) and showed recurrent pathogenic variants in *TP53*, *NOTCH1, BIRC3*, *DNMT3A*, *MYD88*, *SRSF2*, and others, with *TP53* variants clustering with del(17p) or complex genomes (Table 2).

### 3.7. OGM-Negative Cases

Nine patients (18%) had no oncogenic structural variants detected by OGM, yet several harbored clinically significant sequence-level alterations detectable by NGS. For example, (S19) carried a tier 1 pathogenic *SRSF2* missense variant (p.Pro95Leu), while (S47) had a tier 2 *DNMT3A* splice-site variant (c.1015-2A>G). In other OGM-negative patients (e.g., (S1), (S3), (S16), (S18), (S25), and (S28)), all genomic assays were negative, and disease remained indolent (Table 2). Together, these findings highlight that negative results may not definitively exclude pathogenic sequence variants or sub-clonal cytogenetic changes and highlight the complementary roles of cytogenomics and molecule sequence-level alterations in characterizing the genomic spectrum of CLL.

### 3.8. IGHV Somatic Hypermutation Status and Relationship to OGM Profiles

IGHV status was available in 37/50 patients (74%), of whom 21/37 (56.8%) were IGHV-mutated and 16/37 (43.2%) were IGHV-unmutated, across the entire cohort (Table 2). IGHV-mutated cases were enriched among patients with isolated or structurally simple del(13q) or OGM-negative genomes, including several with focal del(13q) long TTFT (e.g., (S5), (S22), (S31), and (S41)) and multiple OGM-negative, clinically stable cases; consistent with indolent biology [8]. In contrast, IGHV-unmutated status frequently accompanied high-risk OGM profiles as noted above, all associated with shorter TTFT. Notably, some OGM-negative but clinically progressing patients (e.g., (S42), (S47)) had unmutated IGHV, whereas two patients with mutated IGHV nevertheless showed poor-risk OGM findings (*RB1*-spanning del(13q) with additional lesions (S8, S20)). This suggests that the IGHV status and the cytogenetic and genomic work-up provided important layers of prognostic information [7,8].

### 3.9. TTFT Analysis and Clinical Correlation

TTFT data were available for seventeen patients and ranged from 0.3 to 192 months, mirroring the heterogeneity of OGM-defined risk categories (Table 3, Figure 2). Cases with adverse structural features, such as del(17p)/*TP53* with complex multichromosomal structural rearrangements ((S34), (S35), and (S44)) and double-*IGH* rearrangements as noted above, clustered at the shortest TTFT intervals (0.3–41 months, Figure 3). In contrast, patients with isolated focal del(13q) or structurally simple genomes had TTFT exceeding 90–190 months, in line with modern integrated models that link genomic complexity with more aggressive disease [7,8,18,19,20,21]. Several long TTFT cases harbored multiple OGM-detected abnormalities that were focal, low-level, or lacked established high-risk features, such as del(17p), or extensive multichromosomal complexity, underscoring that not all structural alterations confer equivalent clinical risk.

**Figure 5:**
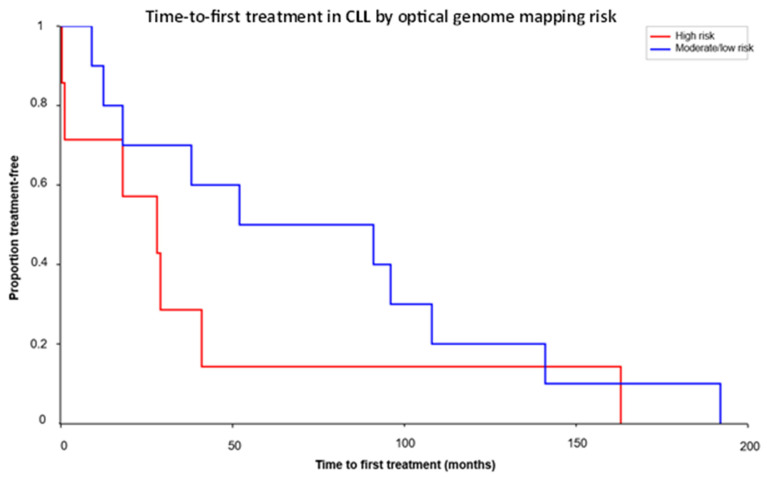
Time-to-first treatment by OGM risk. Kaplan–Meier-style curves showing the proportion of patients (Y-axis) remaining treatment-free in months (X-axis) for OGM-defined high-risk versus moderate-/low-risk genomic groups in the subset with available TTFT.

When OGM-defined high-risk genomes (del(17p), double-IGH rearrangements, and/or complex multichromosomal changes) were compared with all other moderate-/low-risk genomes, median TTFT was 28.0 months in the high-risk group versus 71.5 months in the combined moderate-/low-risk group (Table 3). A nonparametric Mann–Whitney test showed a nonsignificant difference between groups (U ≈ 21.5, *p* ≈ 0.20); however, a trend toward earlier treatment in high-risk patients clearly aligned with the separation of the Kaplan–Meier curves (Figure 5). These findings support a directional association between OGM-defined genomic complexity and earlier treatment requirement. However as noted above (i) the TTFT analyses are based on a smaller subset of patients from the larger cohort described (ii) confidence intervals around group estimates are wide, (iii) *p* values are interpreted descriptively, and (iv) the observed association between genomic complexity and shorter TTFT should be regarded as hypothesis-generating and requiring validation in larger, prospectively collected cohorts.

Notably, several patients who were OGM-negative or lacked tier 1 or tier 2 OGM-detected structural variants (S42, S47, S24, and S29) nevertheless demonstrated early or intermediate TTFT. Review of integrated genomic data (Table 2) showed that these cases were enriched for IGHV-unmutated status and/or pathogenic sequence-level alterations detected by NGS, including *NOTCH1* (S24, S29) and *DNMT3A* (S47). These observations indicate that, in the absence of overt structural complexity, immunogenetic and sequence-level drivers can influence treatment timing, underscoring the complementary role of OGM within a multimodal genomic risk-stratification framework.

## 4. Discussion

In this genome-wide chromosomal structural variant analysis of fifty CLL patients, OGM effectively resolved deletions, balanced and unbalanced rearrangements, multichromosomal copy-number complexity, and rare translocations. By integrating OGM with IGHV status and NGS mutational profile, the study refined diagnostic classification and prognostic stratification, providing a multilayered view of CLL biology.

### 4.1. Structural Lesions and Genomic Complexity

Consistent with the prior cytogenetic literature, del(13q) was the most frequent abnormality, detected in 58% of cases and accounting for the majority of OGM-identified deletions. OGM extended beyond the binary resolution of FISH by defining deletion size and gene content, distinguishing focal 13q14 lesions from larger *RB1*-spanning deletions. Focal del(13q), often in IGHV-mutated cases, was associated with long TTFT and indolent disease, reaffirming its favorable prognostic profile [4,5,7,8]. In contrast, *RB1*-type deletions frequently co-segregated with additional high-risk SVs and, in some instances, IGHV-unmutated status, aligning with evidence that *RB1* loss may contribute to genomic instability and poorer outcomes.

OGM also clarified the broader spectrum of high-risk structural variants, including del(11q)/ATM, *BIRC3* disruption, del(17p)/*TP53*, trisomy 12, and extensive multichromosomal copy-number alterations, all previously associated with inferior survival, chemoresistance, or early relapse [21,22,23,24,25]. Many lesions that appeared “isolated” by FISH were embedded within complex genomes when assessed by OGM.

### 4.2. Diagnostic and Clinical Impact of OGM

OGM uniquely detected multiple clinically meaningful translocations and complex rearrangements. The double-hit IGH case (S10), harboring concurrent *IGH*::*BCL11A* and *IGH*::*BCL3* rearrangements, likely reflects a germinal-center-like transcriptional program with NF-κB activation, concordant with prior mechanistic studies and explaining the highly aggressive clinical behavior [26,27]. Although t(14;18)(*IGH*::*BCL2*) is characteristic of follicular lymphoma and rarely seen in CLL/SLL, WHO recognizes it as a documented but uncommon exception; the two *IGH*::*BCL2*-positive, trisomy 12 cases in this cohort (S45, S48) fit this pattern and support their classification as true CLL with atypical cytogenetics. Standard CLL FISH panels typically do not target many of these *IGH* partners and cannot survey genome-wide translocation patterns. Karyotype analysis would also not inform on genic content and, depending on the quality of the chromosomes recovered, may not fully reveal the cytoband breakpoint based on banding pattern alone. Using the current work-up, we reveal such structural drivers alongside complex CNCs. qwq

OGM uncovered rare immunoglobulin locus rearrangements and their partner genes, as described above. This raises important questions about their clinical implications. Prior large series show that *IGH* translocations occur in a small minority of CLL cases and encompass biologically heterogeneous partners: patients with *IGH*::*BCL2* often display trisomy 12 and atypical morphologic or immunophenotypic features and may have outcomes comparable to low-risk FISH subsets, whereas *IGH*::*BCL3* rearrangements are more consistently linked to higher risk scores and earlier need for therapy [28,29]. Double or multiple productive *IGH* rearrangements likewise appear infrequent and may indicate coexistence of distinct B-cell populations, but robust data on their independent prognostic effect are lacking. At present, therefore, detection of these rare *IGH*-driven events by OGM should primarily prompt careful diagnostic review in the context of accompanying aberrations (e.g., *TP53* disruption, genomic complexity), and consideration of closer clinical surveillance, rather than mandating a specific therapeutic approach on their own. Larger, systematically annotated series will be required to determine when such rearrangements should be incorporated formally into risk models or treatment algorithms.

### 4.3. Integrated Genomic Risk and TTFT

NGS identified recurrent alterations in *TP53*, *NOTCH1*, *BIRC3*, *MYD88*, *DNMT3A*, and other genes, mirroring patterns reported in large sequencing cohorts. These variants exhibited expected combinatorial associations, such as *TP53* with del(17p), *NOTCH1* with trisomy 12, and *BIRC3* with del(11q) [15,25,30,31,32,33,34]. IGHV status added another layer of context: in line with seminal studies, IGHV-unmutated disease was generally aggressive and was enriched among OGM-defined high-risk groups, whereas mutated IGHV predominated in simple del(13q) or OGM-negative genomes.

TTFT trajectories in this cohort mirrored these integrated risk layers as follows: patients with mutated IGHV, isolated focal del(13q), or OGM-negative genomes had the longest TTFT; those with trisomy 12, del(11q), or RB1-type del(13q) had intermediate TTFT; and those with del(17p)/*TP53* and complex or rare structural alterations experienced the shortest TTFT. OGM-negative but NGS- or IGHV-high-risk cases often showed intermediate behavior, reflecting disease kinetics driven more by sequence-level or immunogenetic factors than by large SVs. Together, these observations argue for routine integration of genome mapping-guided digital karyotyping and sequencing-based studies when constructing prognostic models and guiding management.

Beyond prognostication, the integrated chromosomal structural and sequence-level patterns described here have potential implications for therapy selection and clinical trial design in CLL. As precision oncology increasingly emphasizes tailoring treatment to specific molecular mechanisms and the tumor immune microenvironment, genomic alterations such as del(17p)/*TP53* disruption, *NOTCH1* or *BIRC3*-altered trisomy 12, and highly complex multichromosomal SV/CN profiles provide a biologic framework for thinking about which patients may benefit from targeted strategies or novel agents outside of conventional chemoimmunotherapy [35].

Conceptually, our OGM-defined digital karyotypes could be layered onto emerging therapeutic paradigms discussed in broader oncology overviews of newer therapies and future directions, in which molecularly defined subgroups are used to guide both targeted inhibition and modulation of the immune microenvironment [35]. Although the current study was not designed to test treatment effects, these chromosomal structural and sequence-level alterations; used as surrogates for underlying genomic instability or apoptotic resistance, may help identify CLL subsets most likely to benefit from next-generation BCR pathway inhibitors, BCL2 antagonists, or cellular therapies. In particular, they may inform the selection of rational combination strategies that address both tumor-intrinsic lesions and their microenvironmental dependencies.

### 4.4. Strengths, Limitations, and Conclusions

The key strengths of this study include comprehensive whole-genome structural variant profiling by OGM, capturing cryptic balanced rearrangements and multichromosomal CNCs. Additionally, this study reveals several structural patterns in this cohort that appear under-reported in CLL, including a relatively high prevalence of *RB1*-spanning del(13q), multiple low-level cryptic 13q translocations, and multi-hit 13q events. An especially notable double-IGH case (S10) with concurrent *IGH*::*BCL11A* and *IGH*::*BCL3* rearrangements showed highly aggressive clinical behavior. In addition, among OGM-positive cases, multi-chromosomal structural variant changes were detected in 8 of 41 patients (20%), including several cases that would be classified as standard risk by FISH alone. Collectively, these observations support an expanding role for OGM in uncovering clinically relevant structural events that are not captured by conventional diagnostic approaches. Limitations include the retrospective single-institution design, modest sample size (*n* = 50) that restricts subgroup analyses, and incomplete FISH, NGS, and IGHV data in some patients, which may underestimate the full spectrum of correlates. Furthermore, the small sample limits statistical power to detect modest to moderate effect sizes and precludes robust multivariable modeling, so that the observed trends toward earlier treatment in high-risk genomes should not be viewed as definitive evidence of association.

This retrospective, single-institution design also raises the possibility of selection bias, as patients undergoing more extensive genomic testing may differ systematically from the broader CLL population in disease stage, referral patterns, or management strategies. And as noted above, key genomic covariates were incompletely captured, with NGS data available in 58% and IGHV status in 74% of cases, which reduces power for integrative analyses. OGM-informed risk categories remains to be validated in larger cohorts with more complete genomic annotation.

To this end, future studies should systematically integrate OGM-derived measures of genomic complexity into existing prognostic frameworks such as the CLL- International Prognostic Index (CLL-IPI) and formally evaluate whether OGM-enhanced models improve prediction of time-to-first-treatment and survival beyond standard clinical, biochemical, and gene-sequence alteration-based parameters. Prospective, multi-center cohorts with sufficient numbers will be essential in determining whether incorporation of genome-wide structural profiling into CLL-IPI–like tools impacts risk-adapted management algorithms.

Overall, OGM provides a comprehensive, genomic-scale high-resolution view of the structural variant landscape in CLL, revealing deletion architecture, cryptic translocations, and complex multi-hit genomes. When integrated with targeted NGS and IGHV mutational status, OGM yields a robust multilayered genomic profile that closely mirrors clinical behavior and TTFT, supporting its incorporation into contemporary CLL workups and serving as a foundation for future prospective studies evaluating OGM-guided risk stratification and treatment decision-making.

## Figures and Tables

**Table 1:** Baseline characteristics of the study cohort.

Category	Number of Cases
Total Patients	50
Male	25
Female	25
Median Age at Diagnosis (Male)	63
Median Age at Diagnosis (Female)	65
CLL Cases	47
MBL Cases	2
MCL Case (Revised)	1

Monoclonal B-cell lymphocytosis (MBL); mantle cell lymphoma (MCL).

**Table 2:** Comparison of structural variant findings by OGM with corresponding FISH, NGS, IGHV, and clinical impact status in the 50-patient cohort. In this 50 patient cohort, incorporation of OGM, impacted CLL prognostics in 24 of the 50 cases presented, while the remaining retained their original risk categorization. These data highlight that nearly half of patients experienced a refinement of risk stratification when OGM derived structural variant information was added to conventional testing.

Sample ID	Sample Type	OGM Findings	FISH Findings	IGV Mutation Status	NGS Findings	Prognosis Impacted by OGM
S1	BM	Negative	Negative	Unmutated	Negative	No
S2	PB	T1: del(11q)(*ATM*, *BCRL3*), del (13q), T2: del(18p), dup(15q)(*MAP2K1*, *PML*, *IDH2*), reciprocal t(13;21)	del (11q), del(13q)	Unmutated	N/A	Yes
S3	PB	Negative	Negative	N/A	Negative	No
S4	PB	T1: del(13q)	del(13q)	N/A	N/A	No
S5	PB	T1: del(13q)	del(13q)	Mutated	T1: *TP53*	No
S6	BM	T1: t(11;22)(*IGL::CCND1*)	Extra copy of 11q13	Mutated	T2:*DNMT3A*, *NOTCH2*, *BIRC3*, *SMARCB1*	Yes
S7	PB	T1: del (11q)(*ATM*, *BIRC3*)	del(11q)	Unmutated	Negative	Yes
S8	BM	T1: del(13q) (*RB1*)	del(13q)	Mutated	Negative	Yes
S9	PB	T1: del(13q), t(5;13)	del(13q)	Mutated	No T1 or T2	Yes
S10	PB	T1:t(2;14)(*IGH*::*BCL11A*), t(14;19)(*IGH*::*BCL3*), partial copy number gain on chromosome 12	Negative	Unmutated	N/A	Yes
S11	PB	T1: Trisomy 12	Trisomy 12	N/A	N/A	No
S12	PB	T1: del(13q), Trisomy12, T2:de(l1p)(*CSF3R*)	del(13q), Trisomy 12	Mutated	T1:*NOTCH1*	Yes
S13	PB	T1: del(13q)(*RB1*), T2:ins17q23.3(*CD79B*)	del(13q)	Mutated	N/A	Yes
S14	PB	T1: del(13q) (*RB1*),T2: gains of chromosomes 3, 8q(*MYC*), and 18	del(13q)	N/A	N/A	Yes
S15	PB	T1: del(13q)(*RB1*), t(1;13), t(7;13), t(9;13)	del(13q)	N/A	N/A	Yes
S16	PB	Negative	Negative	N/A	N/A	No
S17	PB	T1: Bi and mono allelic del(13q)	del(13q)	Mutated	No T1 or T2	No
S18	PB	Negative	Negative	Unmutated	No T1 or T2	No
S19	BM	Negative	Negative	N/A	T1: *SRSF2*	No
S20	PB	T1: del(13q) (*RB1*), reciprocal translocation between 2p13, 2q36.3, and 13q	del(13q)	Mutated	T1:*NOTCH1*, *BIRC3*	Yes
S21	PB	T1: del(13q)	del(13q)	unmutated	T1:*TP53*	No
S22	PB	T1: del(13q)	del(13q)	Mutated	N/A	No
S23	PB	T1: del(13q)(*RB1*)	del13q	Mutated	N/A	No
S24	BM	No T1 or T2	Negative	Unmutated	T1: *NOTCH1*	No
S25	PB	Negative	Negative	N/A	N/A	No
S26	PB	T1: del(13q), interstitial del(17p) (*TP53*)	del(13q), del(17p)	Mutated	N/A	No
S27	PB	T1: del(13q)	N/A	Mutated	Negative	Yes
S28	PB	Negative	Negative	N/A	N/A	No
S29	BM	No T1 or T2	Trisomy 12 (low-level)	Unmutated	T1: *NOTCH1*	No
S30	PB	T1: Trisomy12	Trisomy 12	Unmutated	N/A	No
S31	PB	T1: de(l13q)	del(13q)	Mutated	N/A	No
S32	PB	T1: del(13q)(*RB1*)	del(13q)	Mutated	N/A	No
S33	PB	T1: del(13q), reciprocal translocation between 13q14 and 21q22.2, T2: CN gain 5q34-q35	del(13q)	N/A	N/A	Yes
S34	PB	T1: del(17p), paracentric translocation/inversion involving 13q14, T2: complex CN change in 12p and 12q	del(17p)	unmutated	T1:*TP53*, *NOTCH1*	Yes
S35	PB	T1: del(13q)(*RB1*), del17p, complex CNC 9p&9q, T2: Complex CNC 2p, 2q, loss 8p	del(17p), del(13q)	N/A	T1:*TP53*	Yes
S36	PB	T1: del(13q)(*RB1*)	del(13q)	Mutated	Negative	No
S37	PB	T1: Trisomy 12, t(18;22)(*IGL::BCL2*)	Trisomy 12	N/A	N/A	Yes
S38	BM	T1: del(11q)(*ATM*&*BIRC3*)(low level), del(13q), Monosomy 13, reciprocal t(13q;14q) (low level)	del(13q)(low level)	Mutated	No T1 or T2	Yes
S39	PB	T1: del(13q)(RB1)	del(13q)	Mutated	Negative	No
S40	PB	T1: Trisomy12, del9p22 (*MLLT3*, *CDKN2A*)	Trisomy12	unmutated	Negative	Yes
S41	BM	T1: del(13q)	del(13q)	Mutated	No T1 or T2	No
S42	PB	Negative	Negative	Unmutated	N/A	No
S43	PB	T1: Trisomy 12, reciprocal t(1q23;13q14), T2: gains of chromosomes 18 and 19	Negative	N/A	N/A	Yes
S44	PB	T1: del(13q), del(17p), T2: t(8:14), CN gain of 2p, 7p, and 18, CN loss 8p, 20q	Extra copy of *IGH*, del(17p), biallelic del(13q)	Unmutated	T1:*TP53*	Yes
S45	PB	T1: Trisomy 12, t(14:18) (*IGH*:*BCL2*)	Trisomy12	Mutated	No T1 or T2	Yes
S46	PB	T1: del(11q)(*ATM*, *BIRC3*), del(13q)	del(11q), del(13q)	Unmutated	N/A	Yes
S47	BM	Negative	Negative	Unmutated	T2: *DNMT3A*	No
S48	PB	T1: trisomy12, del(13q) (*RB1*), t(14:18)(*IGH*:*BCL2*)	extra copy *IGH*, del(13q), trisomy 12	Mutated	T1:*NOTCH1*	Yes
S49	PB	T1: del(13q) (*RB1*), T2: t(1:13)	del(13q)	Unmutated	T1: *NOTCH1*	Yes
S50	PB	T1 Trisomy 12, del(13q)	trisomy 12, del(13q)	Mutated	T1: *NOTCH1* T2: *MYD88*	No

“N/A” indicates that no result was available because testing was not performed, was not ordered clinically, or data were not accessible in the medical record. T1 and T2 refer to tier 1 pathogenic or tier 2 likely pathogenic, respectively. S1–S50 indicate samples identifiers 1–50 in this cohort. ‘PB’ and ‘BM’ refer to peripheral blood and bone marrow respectively. CNC is annotated as complex copy number change(s).

**Table 3:** Time-to-first treatment (TTFT) in 17 CLL patients with available treatment data.

Case	TTFT(Months)	OGM Results
S44	0.3	T1: Trisomy 12, reciprocal t(1q23;13q14), T2: gains of chromosomes 18 and 19
S10	1.1	T1:t(2;14)(*IGH*::*BCL11A*), t(14;19)(*IGH*::*BCL3*), partial copy number gain on chromosome 12
S8	9	T1: de(13q) (*RB1*)
S42	12.4	Negative
S2	18	T1: del(11q)(*ATM*, *BIRC3*), del(13q), T2: del(18p), dup(15q22)(*MAP2K1*, *PML*, *IDH2*), reciprocal t(13;21)
S29	18	No T1 or T2
S34	28	T1: del(17p), paracentric translocation/inversion involving 13q14, T2: complex CNC in 12p and 12q
S7	29	T1: del(11q)(*ATM*, *BIRC3*)
S24	38	No T1 or T2
S35	41	T1: del(13q)(*RB1*), del(17p), complex CNC 9p&9q, T2: Complex CNCs 2p, 2q, loss 8p
S47	52	Negative
S38	91	T1: del (11q)(*ATM*&*BIRC3*)(low level), del(13q), Monosomy 13, reciprocal t(13q;14q) (low level)
S49	96	T1: del(13q) (*RB1*), T2: t(1:13)
S20	108	T1: del(13q) (*RB1*), reciprocal translocation between 2p13, 2q36, and 13q
S41	141	T1: del(13q)
S45	163	T1: Trisomy 12, t(14:18) (*IGH*::*BCL2*)
S33	192	T1: del(13q), reciprocal translocation between 13q14 and 21q22.2, T2: CN gain 5q34-q35

## Data Availability

Data supporting the findings of this study are available from the corresponding author upon reasonable request.
